# Molecular genetics of non-syndromic deafness

**DOI:** 10.1016/S1808-8694(15)31313-6

**Published:** 2015-10-20

**Authors:** Vânia B. Piatto, Ellen C.T. Nascimento, Fabiana Alexandrino, Camila A. Oliveira, Ana Cláudia P. Lopes, Edi Lúcia Sartorato, José Victor Maniglia

**Affiliations:** 1 Professor, Department of Anatomy (FAMERP), Pediatrician (Hospital de Base – FUNFARME); 2 Medical school undergraduate – FAMERP; 3 Biomedics, Ph.D. in Medical Sciences under course – UNICAMP; 4 Biomedics, Ph.D. in Medical Sciences under course – UNICAMP; 5 Professor, Department of Anatomy – FAMERP; 6 Researcher, Laboratory of Human Genetics, Center of Molecular Biology and Genetic Engineering (CBMEG) –UNICAMP; 7 Head of the Discipline of Otorhinolaryngology, Executive Director of Medical School, São José do Rio Preto, SP – FAMERP

**Keywords:** deafness, non-syndromic, molecular genetics

## Abstract

One in every 1,000 newborn suffers from congenital hearing impairment. More than 60% of the congenital cases are caused by genetic factors. In most cases, hearing loss is a multifactorial disorder caused by both genetic and environmental factors. Molecular genetics of deafness has experienced remarkable progress in the last decade. Genes responsible for hereditary hearing impairment are being mapped and cloned progressively. This review focuses on non-syndromic hearing loss, since the gene involved in this type of hearing loss have only recently begun to be identified.

## INTRODUCTION

In developed countries, approximately 1/1,000 children have severe or profound hearing loss at birth or during childhood, at pre-lingual stage. About 60% of the cases have hereditary etiology, 30% of the cases are acquired, and 10% are idiopathic. Non-syndromic forms are responsible for 70% of the cases of hereditary etiology and syndromic cases represent 30% of them. Among the forms of heritage, autonomic recessive is the most frequent one (75%-85%), followed by dominant heritage (12-13%) and X-linked or mitochondrial, with 2-3% of the cases of non-syndromic hearing loss.^1^, ^2^

Syndromic or autosomal dominant hearing losses may cause conductive, sensorineural or both losses. Conversely, pre-lingual non-syndromic cases of autosomal recessive losses are almost always sensorineural^1^, ^2^. Syndromic abnormalities caused by rubella, toxoplasmosis, cytomegalovirus, syphilis or use of drugs in gestational period may cause hearing disorders, which are congenital but not genetically-based, such as other syndromic forms of hearing loss. Many of these syndromes have already been described, and genes have been mapped and cloned.^1^

Some of the identified genes that cause syndromic forms are also responsible for isolated forms of hearing loss. There does not seem to be a direct correlation between this type of mutations and the association with syndromic or non-syndromic hearing loss. Moreover, analyses of phenotypes together with mutations in some affected families by Pendred or Usher syndrome, for example, revealed that mutating gene of these syndromes may also cause non-syndromic hearing loss. In such cases, it is highly likely that modifying genes are contributing factors^3^.

The purpose of the present study was to review some genes described to present that, if mutating, cause the most varied forms of non-syndromic hearing loss: autosomal recessive, dominant, X-linked and mitochondrial. We selected articles that were part of OMIM database using MedLine, and the search mechanism used key words such as *“deafness”* and *“non-syndromic”*. Selected articles were those that provided the more recent information on genes involved and their respective proteins, sites of expression in the cochlea and audiological clinical picture.

## LITERATURE REVIEW

### Nomenclature of Non-syndromic Hearing Losses

Different chromosome sites of non-syndromic forms of genetic deafness are named under the acronym DFN (from English *deafness*) followed by letters A or B, meaning autosomal dominant transmission (DFNA) and recessive transmission (DFNB), respectively. When using DFN isolated, it is X-linked deafness. After the letters, there is a whole number, indicating the order of gene discovery.^2^

### Hearing Physiology

To understand the consequences of gene mutations that regulate the hearing process, we have to know about normal cochlear physiology^4^. After sound stimulus, sound mechanical energy is converted into electrical signal (mechanic-electrical transduction) in outer hair cells of the cochlea. On the apical surface of these hair cells, there are specialized microvillus - stereocilia - comprising corium with actin and external recover of myosin, which oscillate in response to sound, which is secondary to oval window stapes movement, which moves the liquid that surrounds hair cells. The deflection of neighboring stereocilia opens the transduction channels in them, allowing inflow of potassium from endolymph to both hair cells, causing depolarization of cell membrane and activating calcium channels on the basolateral surface of the membrane, which are sensitive to voltage modifications. There is subsequent inflow of calcium causing release of vesicles that contain neurotransmitters, in the synaptic endings of 8th cranial nerve. Thus, after sound stimulus, hair cells are hyperpolarized with high concentration of intracellular potassium. So that new excitation is made possible, potassium has to be removed. This movement of potassium ions from hair cells to cochlear supporting cells, going back to the endolymph, is made by intercellular and specialized communications, the so-called communicating junctions or gap junctions that exist between supporting cells, fibrocytes of spiral ligament and spiral limbus.^4^

### Molecular Genetics of Non-Syndromic Hearing Loss

**Genetically-codified proteins with expression on cochlear hair cells**:

1) Non-conventional myosin proteins:
  Non-conventional myosin proteins form a family that is divided into 16 classes, found in most non-muscular cells^5^. They are smaller than muscle myosin and for this reason they are called mini-myosin. These motor proteins form filaments that move in actin filaments using energy generated by ATP hydrolysis. In the cochlea, they have been implied in the formation and movements of expansion of cytoplasmatic membrane, synaptic vesicle movements and transduction signals of outer and inner hair cells.^5^
  a) Myosin VIIA: it is expressed in inner ear outer hair cells and in a great variety of epithelial cells that present apical microvilosities, in addition to retina photoreceptor cells. In the cochlea, the protein is present along the stereocilia, close to the junction between hair cells and supporting cells and present in the synaptic region, Gene MYO7A, located in chromosome 11 (11q13.5), has 49 exons that codify non-conventional myosin protein VIIA (2215 amino acids).^6^ Mutations in the gene cause structural defects of the protein and consequent affections in auditory function, responsible for non-syndromic firms of hearing loss, one of profound recessive autosomal transmission – DFNB2, comprising different grades of vestibular dysfunction and variable age of onset, and another one that is autosomal dominant – DFNA11, whose onset takes place only after complete speech acquisition and causes progressive hearing loss.^6^
    When the mutations cause also retina cell abnormalities, phenotypic picture is characterized as Usher syndrome. The chromosomic site for one of the genetic types of Usher syndrome – USH1B was also mapped in the same region of chromosome 11, responsible for 75% of the cases of USH type 1^7^. Usher syndrome is the more frequent cause of hearing loss associated with blindness and vestibular pathology since childhood. Studies on mutation of gene MYO7A that causes DFNB2, DFNB11 and Usher1B were the first ones to show that one single gene could determine both forms of hearing loss, syndromic and non-syndromic^7^.
  b) Myosin XV: non-conventional protein (3,530 amino acids) codified by gene MYO15 with at least 50 exons and located on chromosome 17 (17p11.2). In the inner ear, the expression of this gene seems to be restricted to hair cells, on the cuticular plaque. Mutations of this gene determine DFNB3.^8^
  c) Myosin VI: gene MYO6 (32 exons), located at chromosome 6 (6q13), codifies non-conventional protein myosin VI (1262 amino acids), concentrated on cuticular plaque of hair cells^1^. Mutations determine DFNA22 and DFNB37, characterized by progressive hearing loss, post-lingual, which starts during childhood (8 to 10 years to start symptoms, 6 to 8 years for onset of audiometric affections), progressing to profound level at the age of 50 anos.^9^
  d) Myosin III: non-conventional protein recently described in a Jewish family of Mosul, in Iraq, codified by gene MYO3A (10p11.1).^10^ Mutations determine DFNB30, characterized by bilateral progressive hearing loss that affects primarily high frequencies, starting on the second decade, and at age 50 years, it reaches severe level in high and medium frequencies and moderate level in low frequencies.^10^
2) Harmonin: gene site, if mutant, causes DFNB18, and was mapped at chromosome 11 (11p15.1), at the same gene location as USH1C (Usher Syndrome Type IC – 11p15.1).^7^, ^11^ Gene USH1C (28 exons) codifies a protein that contains domain PDZ, denominated harmonin. At the cochlea, harmonin is restricted to hair cells, in which it is present in the cellular body and stereocilia. In patients with pre-lingual and severe DFNB18, mutation of gene USH1 has been recently detected, located in an alternative exon present in the transcription to the inner ear, but not to the retina transcription^11^. Functional characterization of domain corresponding to harmonin protein provides the understanding of the pathogenesis of DFNB18 and USH1C syndrome.^7^
3) Villin: protein that belongs to the molecule that contains PDZ domain; it acts as an organizer of submembranous molecular complexes that control and coordinate polymerization of actin for the growth of membrane in stereocilia of inner and outer hair cells. Gene villin (9q32-q34), with 12 exons, codifies protein of the same name, with 465 amino acids and if mutant they are responsible for pre-lingual profound DFNB31. Protein villin is similar to protein harmonin because it shares 95% of its three PDZ domains.^12^
4) Cadherin-23: it belongs to the family of transmembrane proteins, dependent on Ca^2+^ ions, with over 20 different members, making part of a molecular structure of intercellular adhesion junctions or zones of adhesion (zonnula adherens). Chromosome sites for DFNB12 (10q21-q22)^13^ and Usher syndrome Type I (USH1D – 10q)^7^ were mapped in chromosome 10. Gene CDH23, with 69 exons codifies protein cadherin-23 (3354 amino acids) expressed in both cochlear hair cells, promoting strong adhesion between each of their types, maintaining polarization of plasma membrane depending on occlusion junctions (claudin-14 protein) and cytoskeleton. Mutations of gene CDH23 were detected in families with DFNB12, which presented pre-lingual profound hearing loss^13^. Conversely, only deletions or displacements were found in patients with USH1D.^7^ Therefore, the type of mutation can have a crucial role in phenotypic expression.
5) Diaphanus-1: it belongs to family of proteins related to formins, involved in cell polarization and cytokinesis. Gene DIAPH1 or HDIA1 (26 exons), located at chromosome 5 (5q31), codifies protein diaphanous-1 (1252 amino acids),^14^ homologous to protein diaphanous of *Drosophila*. At the cochlea, the protein is found in hair cells and external supporting cells, but in small concentrations. Gene mutations affect the cytoskeleton of actin in outer hair cells and cause DFNA1, described in a family in Costa Rica, in which they located the first affected ancestral named Monge.^14^ It is characterized by progressive hearing loss that at first affects low frequencies (Konigsmark syndrome, by identification of three families with hearing loss and this audiological pattern). At the age of 40 years, approximately, hearing loss reaches severe level in all frequencies.^14^
6) KCNQ4: gene KCNQ4, with 14 exons, mapped in chromosome 1 (1p34), codifies a protein subunit of family KCNQ of potassium channels, protein KCNQ4 (695 amino acids). In the cochlea, channels KCNQ4 are expressed not only in outer hair cells, but also in inner hair cells, whose main function is to promote the outflow of potassium from the cells to supporting cells.^15^ Mutations of this gene were identified in families affected by progressive hearing loss – DFNA2, starting at adolescence or at the age of 20 years, and preferably involving high frequencies, becoming profound within 10 years.^15^
7) Otoferlin: gene OTOF, with 48 exons, codified protein otoferlin (1977 amino acids) located at chromosome 2 (2p22-p23), whose mutation determines DFNB9, characterized by pre-lingual profound hearing loss involving all frequencies.^16^ Protein otoferlin is expressed in inner hair cells and it is involved in the fusion, triggered by calcium, of synaptic vesicles with the plasma membrane, releasing glutamate neurotransmitter to the afferent innervation system to take the sound message codified by inner hair cells in the form of electrical impulses to the central auditory areas.^16^
8) POU4F3: A deletion of only 8 base pairs was the mutation found in gene POU4F3 (2 exons), located in chromosome 5 (5q31), determining DFNA15, starting between 18 and 30 years, progressive, and which reaches moderate to severe level at the age of 50, approximetaly.^17^ Gene POU4F3 codifies transcription factor of the same name (338 amino acids), belonging to the family of proteins of domain POU. In both hair cells in the cochlea, gene POU4F3 seems to express the migration of the same layers of supporting cells for the hair cell layer of the lumen in addition to their maturation and survival.^17^

**Proteins genetically codified with expression on cochlear non-sensorial cells**:

1) Protein connexin:
  Protein connexin is the structural component of intercellular gap junctions, which are responsible for flow of potassium of supporting cells for fibrocytes of spiral ligament and spiral limbus back to endorphin, after it has been out from the hair cells.
  a) Connexin 26: in 1997, gene connexin 26 (13q11-12) was discovered, whose mutations caused DFNA3 and DFNB1.^18^ It has taken to the assumption that gene Cx26 or GJB2, with only one exon, codifies protein connexin 26 (226 amino acids), which can be responsible for both forms of hearing loss. Hearing loss is characterized by being pre-lingual, non-progressive, profound, with high threshold values in all frequencies.^18^
  b) Connexin 31: it has not been determined yet if protein connexin 31 (270 amino acids) is present in all gap junctions of the inner ear. The site of Cx31 or GJB3, in chromosome 1 (1p34) is the same for gene KCNQ4, expressed in both hair cells, and if mutant, it causes DFNA2.^19^ Owing to that, mutations of gene Cx31 also cause dominant hearing loss, but even with the expression in different site of gene KCNQ4, both received the same name – DFNA2.
  c) Connexin 30: the gene that codifies connexin 30 (261 amino acids) is located in chromosome 13 (13q12)^20^ and if mutant it causes DFNA3 and DFNB1 (both forms also caused by Cx26). If no mutation is found in gene Cx26 or in heterozygote patients for 35delG, mutations of gene Cx30, by its close relation (about 76% of identical amino acids) and proximity of its chromosomic location to gene Cx26, they may be considered responsible for hearing loss, named similarly to Cx26. This fact is explained, in addition to proximity, by the fact that Cx26 and Cx30 may form heterotopic channels of connexons and they have the same cellular distribution in the cochlea. Therefore, pathophysiological hypotheses concerning hearing loss associated with Cx26 and Cx30 are similar.^20^
2) Pendrin: Protein pendrin (780 amino acids) is codified by gene PDS (21 exons), located at chromosome 7. Mutations in this gene are responsible both by Pendred Syndrome (7q21-34) and DFNB4 (gene SLC26A4 – 7q31).^21^ DFNB4 is characterized by progressive hearing loss and widening of vestibular aqueduct, without thyroid affection. In the mature cochlea, protein pendrin is expressed in prominent spiral cells and cells adjacent to external spiral sulcus.^21^
3) Claudin-14: gene CLDN14, located in chromosome 21 (21q22), codified protein claudin (239 amino acids), one of the components of gap junctions or tight junctions.^22^ Occlusion or gap junctions limit the passive diffusion of ions and small molecules through intercellular space, in addition to maintaining cellular polarity together with cytoskeleton and adhesion junctions (cadherin-23 protein). In the cochlea, the gene is expressed in hair cells and supporting cells. Mutations of this gene are responsible for DFNB29.^22^
4) Cocline: protein cocline (550 amino acids) is codified by COCH (11 exons), located in chromosome 14 (14q12-q13). In the cochlea, the gene is expressed in spiral ganglion and extracellular matrix especially spiral limbus, spiral ligament and bone spiral lamina.^23^ Mutations are responsible for DFNA9, which starts between the ages of 20 and 30 years, approximately. Initially, it is profound in high frequencies with variable progression to anacusis at the age of 40-50 years. The spectrum of vestibular involvement varies from absence of symptoms to presence of vertigo and vestibular hypofunction. Mutations of gene COCH may be one of the genetic factors that contribute to symptoms of Mèniére Disease, and this hypothesis should be considered in patients with symptoms of the disease.^23^ Histopathological analyses of temporal bone in patients with DFNA9 showed deposits of mucopolysaccharides in cochlear and vestibular nerve channels. These findings suggest that deposits may take to degeneration of inner ear neural fibers, causing hearing loss.^1^
5) EYA4: gene EYA4 (21 exons), a member of family EYA homologous to Drosophila eyes absent (regulator of ocular development of *Drosophila*), was mapped in chromosome 6 (6q22.3-23.3) that codifies protein EYA4 (639 amino acids).^24^ Genes EYA are expressed in different tissues at the beginning of embryogenesis, and even though each gene EYA has one single pattern of expression, there is major overlapping, that is, EYA1 and EYA4 are both expressed in the optic vesicle and in its derivates. Differently from the phenotype resulting from mutations in gene EYA1 (Brachio-Oto-Renal Syndrome), no congenital anomaly is part of phenotype of DFNA10, characterized by progressive hearing loss whose onset is from 2nd to 5th decades, progressing from severe to profound loss. Losses start in medium frequencies and eventually involve low and high frequencies.^24^
6) POU3F4: Gene POU3F4 (1 exon), mapped in chromosome X (Xq21.1) is responsible for transcription regulating elements.^25^ Expression of gene POU3F4 in the development of the inner ear is restricted to the mesenchyma. Transcription starts when mesenchyma is condensed to originate the optical capsule and protein POU3F4 (361 amino acids), remaining in the nuclei of mesenchymal cells. They then migrate to cavitary regions of temporal bone to form scala vestibularis, scale tympany and internal acoustic canal. In adult cochlea, the gene is expressed in the fibrocytes of spiral ligament. Mutations in these genes cause DFN3, the first non-syndrome form that is X-linked. It has a unique phenotype because affected patients present conductive hearing loss that is probably caused by fixation of stapes, together with progressive profound hearing loss.^25^

**Genetically codified proteins with expression on tectorial membrane**:

1) Collagen XI (alpha2 chain): Collagen XI protein, codified by gene COL11A2 (62 exons) located in chromosome 6, is one of the components of tectorial membrane.^26^ It is an acellular membrane comprising many different types of collagen (II, V, IX, XI), non-collagen proteins and proteoglicans, and it is involved in deflection of ciliary bundle of cochlear outer hair cells, immediately after sound stimulus.^5^ Mutations of gene COL11A2 cause both DFNA13 (6p21), such as Stickler syndrome Type 2 (STL2 – 6p21.3, progressive myopia, early vitreo-retina and articular degeneration, facial hypoplasia, deafness). DFNA13 is characterized by post-lingual progressive loss starting from the 2nd and 4th decades of life and there are some rare patients with vestibular disorders.^26^
2) Alpha-tectorine: many different types of cells synthesize alpha-tectorine protein during development of the inner ear. Due to sequence of DNA in TECTA gene, it is assumed that tectorine protein is synthesized from a precursor adjacent to plasma membrane, via glycosil-phosphatidyninositol, released from the membrane by proteolytic cleavage of precursor. Gene TECTA (23 exons), located in chromosome 11, codifies alpha-tectorine protein (2155 amino acids) and it is one of the components of tectorial membrane.^27^ Mutations in gene cause two forms of autosomal dominant hearing loss (DFNA8 and DFNA12 – 11q22-24, both pre-lingual and they may be progressive and non-progressive) and an autosomal recessive form (DFNB21 – 11q, pre-lingual, severe to profound).^27^ Phenotypic expression may range depending on the occurrence of impaired alleles, because s Swiss family was identified as possibly being a digenic penetrance of hearing loss, involving location of DFNA12, in chromosome 11, and location DFNA2 in chromosome 1.^28^

**Forms of hearing loss caused by mitochondrial DNA affections**:

Diseases related to mitochondrial DNA are transmitted to both genders, only by the mother, and they may be syndromic or non-syndromic. Mitochondral DNA codifies 13 RNA messenger (RNA-m), 2 RNA ribosomic (RNA-r) and 22 RNA-transporters (RNA-t).

Mutation 1555A->G was detected in mitochondrial gene 12S rRNA in patients with family hearing loss and also in isolated cases of hearing loss induced by the use of aminoglycoside antibiotics.^29^ This mutation takes susceptible subjects to hearing loss after treatment with aminoglycosides in concentrations that would not normally affect hearing.^29^

To present, other described non-syndromic mitochondrial mutations that cause hearing loss followed or not by other affections are located in gene RNA-transporter – gene tRNA Ser (UCN): 7445A->G = keratoderm palmoplantar; 7472insC = neurological dysfunction – ataxia, dysarthria and myoclonus; 7510T->C and 7511T->C = only hearing loss. Syndromic mitochondrial mutations can also be located in RNA-t, causing hearing loss associated with neuromuscular syndrome or diabetes mellitus. Recent studies suggested that mitochondrial mutations, such as deletions del4977 pb, del4834 pb and del3867 pb may be responsible for family cases of presbyacusis.^1^, ^3^

### Otosclerosis

Hearing loss caused by clinical otosclerosis has prevalence of 0.2 to 1% among Caucasian adults. The mean age of onset is 3rd decade and 90% of affected patients are below the age of 50 years at the time of diagnosis. Conductive hearing loss is developed when the focus invades stapedial-vestibular articulation, on the oval window, interfering with free movement of stapes. Profound sensorineural hearing loss, reaching all frequencies, may also be present, characterizing cochlear otosclerosis, in about 10% of affected subejcts.^1^, ^3^ Location of OTSC1, OTSC2 and OTSC3, respectively, in chromosomes 15 (15q26.1-qter), 7 (7q34-q36) and 6 (6p21.3-22.3) were identified in families with autosomal dominant transmission for otosclerosis. However, in most cases, etiology remains unknown.^30^

## DISCUSSION

The fact that one same mutation leads to different clinical presentations may be the indication that the knowledge of molecular genetics has not reached the details of auditory dynamics yet, as well as the myriad of neurological abnormalities involved. However, it seems to move towards that. New mutations are described, new genes are cloned and mapped, and there are about 34 genes already identified to form recessive autosomal non-syndromic forms, 40 genes for dominant autosomal forms, 8 for X-linked forms, and 2 genes for mitochondrial heritage.^1^, ^2^

Despite the significant advances in understanding molecular basis of hearing loss, precise identification of genetic cause still presents some difficulties, owing to phenotypical variation. First we have to rule out non-genetic causes, then syndromic causes, and then look for non-syndromic causes.

Most non-syndromic recessive autosomal forms cause pre-lingual loss that is severe to profound and not associated with radiological findings. Exception to this rule are DFNB2 (MYO7A)^6^, DFNB8/10 (TMPRSS3) and DFNB16 (STRC)^1^, ^3^ in which age at onset may occur in later phases of childhood; DFNB4 (SLC26A4)^21^ in which there may be dilation of vestibular aqueduct and endolymphatic sac, and DFNA9 (COCH)^23^ that may be associated with degeneration of cochlear nerve fibers by deposits of mucopolysaccharides. Not very frequent phenotypes in autosomal dominant forms of hearing loss include low frequency hearing loss in DFNA1 (HDIA1) and DFNA6/14/38 (WFS1),^1^, ^3^ medium frequency loss in DFNA8/12 (TECTA)^27^ and DFNA13 (COL11A2)^26^ and vestibular signs and symptoms in DFNA9 (COCH)^23^ and sometimes in DFNA11 (MYO7A).^6^ Owing to the great variety of genes involved, and in view of costs, assessment should be the most specific possible, maybe based on clinical picture. Expectations concerning results and conclusion in relation to them should be very careful.

Otorhinolaryngologists, pediatricians and geneticists should be aware of this phenotypical variety and especially that DFNB1 is the most frequent form of non-syndromic recessive autosomal hearing loss; molecular investigation should be made in such cases, reducing the costs of complementary tests normally requested for the investigation of patients with hearing loss.

Facility and benefits of genetic tracking, especially for mutations that cause DFNB1, should make it an important public health issue so that determinations of early diagnosis of hearing loss can be properly established. Molecular tests can not help all children with hearing loss and it is not reasonable to wait for these tests to replace already existing screening programs. Whether or not screening programs with acoustic otoemissions and brainstem evoked audiometry should include molecular tests for DFNB1 is another different issue.

Genetic counseling of families whose parents have normal hearing and one single hearing child has been very difficult owing to nonexistence of genetic tests to identify specific mutations, especially in developing countries. In most cases, considering the important role of environmental causes of pre-lingual hearing loss, it is difficult to recognize whether hearing loss is of genetic origin. It is essential to inform healthcare professionals, the general population, and the hearing impaired population about genetic advances, and to train professionals on genetic counseling.

Genetic tests for hearing loss are a reality because they have changed the assessment pattern of patients with hearing impairment and should be used by physicians for diagnostic purposes. In the next years, there will certainly be an expansion in the role of these tests and counseling will not be limited to reproductive results. Even though tests may be confusing for medical professionals that are not used to them, in daily practice, they are an important part of medical care. New findings and technologies will expand and enhance complexity of these tests and it will be on Otorhinolaryngologists and Pediatricians to get familiar with recent discoveries and include them in their investigation protocols - the genetic tests.

Reaction to sounds is the first sign that a child has his auditory capacity preserved. Owing to delay in speech acquisition, absence of reaction to sounds or other disorders, parents are the first ones to suspect of hearing loss. The delay between suspicion and diagnosis reduces significantly the possibilities of rehabilitation, because if intervention does not take place early, it will cause communication deficits that have significant morbidity, which can be manifested by paucity of social activities and professional opportunity losses. Conversely, it is surprising that some parents and even some professionals hesitate to accept hearing loss, considering it as unimportant, translating lack of knowledge about the importance of auditory function for the development of conceptual processes that support human's reasoning and speech.
